# Structural insights into how Prp5 proofreads the pre-mRNA branch site

**DOI:** 10.1038/s41586-021-03789-5

**Published:** 2021-08-04

**Authors:** Zhenwei Zhang, Norbert Rigo, Olexandr Dybkov, Jean-Baptiste Fourmann, Cindy L. Will, Vinay Kumar, Henning Urlaub, Holger Stark, Reinhard Lührmann

**Affiliations:** 1 Department of Structural Dynamics, MPI for Biophysical Chemistry, Göttingen, Germany; 2 Cellular Biochemistry, MPI for Biophysical Chemistry, Göttingen, Germany; 3 Bioanalytical Mass Spectrometry, MPI for Biophysical Chemistry, Göttingen, Germany; 4 Bioanalytics Group, Institute for Clinical Chemistry, University Medical Center Göttingen, Göttingen, Germany

**Keywords:** RNA splicing, Cryoelectron microscopy

## Abstract

During the splicing of introns from precursor messenger RNAs (pre-mRNAs), the U2 small nuclear ribonucleoprotein (snRNP) must undergo stable integration into the spliceosomal A complex—a poorly understood, multistep process that is facilitated by the DEAD-box helicase Prp5 (refs. ^1–4^). During this process, the U2 small nuclear RNA (snRNA) forms an RNA duplex with the pre-mRNA branch site (the U2–BS helix), which is proofread by Prp5 at this stage through an unclear mechanism^5^. Here, by deleting the branch-site adenosine (BS-A) or mutating the branch-site sequence of an actin pre-mRNA, we stall the assembly of spliceosomes in extracts from the yeast *Saccharomyces cerevisiae* directly before the A complex is formed. We then determine the three-dimensional structure of this newly identified assembly intermediate by cryo-electron microscopy. Our structure indicates that the U2–BS helix has formed in this pre-A complex, but is not yet clamped by the HEAT domain of the Hsh155 protein (Hsh155^HEAT^), which exhibits an open conformation. The structure further reveals a large-scale remodelling/repositioning of the U1 and U2 snRNPs during the formation of the A complex that is required to allow subsequent binding of the U4/U6.U5 tri-snRNP, but that this repositioning is blocked in the pre-A complex by the presence of Prp5. Our data suggest that binding of Hsh155^HEAT^ to the bulged BS-A of the U2–BS helix triggers closure of Hsh155^HEAT^, which in turn destabilizes Prp5 binding. Thus, Prp5 proofreads the branch site indirectly, hindering spliceosome assembly if branch-site mutations prevent the remodelling of Hsh155^HEAT^. Our data provide structural insights into how a spliceosomal helicase enhances the fidelity of pre-mRNA splicing.

## Abstract (web summary)

The cryo-electron microscopy structure of a newly identified, early spliceosomal complex reveals the mechanism by which the RNA helicase Prp5 enhances the fidelity of the excision of introns from precursor mRNAs.

## Main

**Fig. 1:**
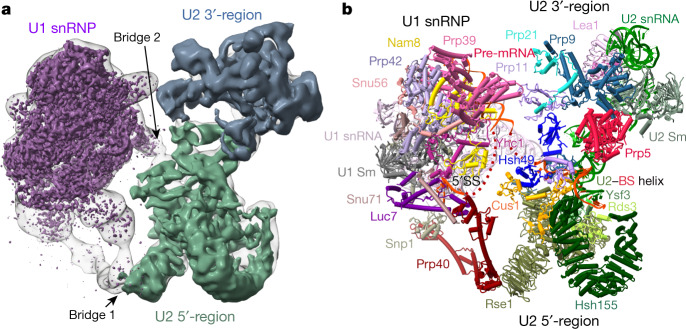
Three dimensional cryo-EM model of the yeast pre-A complex. **a**, **b**, EM density map (**a**) and molecular architecture (**b**) of the *S. cerevisiae* spliceosomal pre-A complex. **a**, Purple, better-resolved U1 density; grey blue and green, better-resolved densities of the 3′- and 5′-regions of U2 snRNP; translucent grey, cryo-EM map of the pre-A complex.

**Extended Data Fig. 1:**
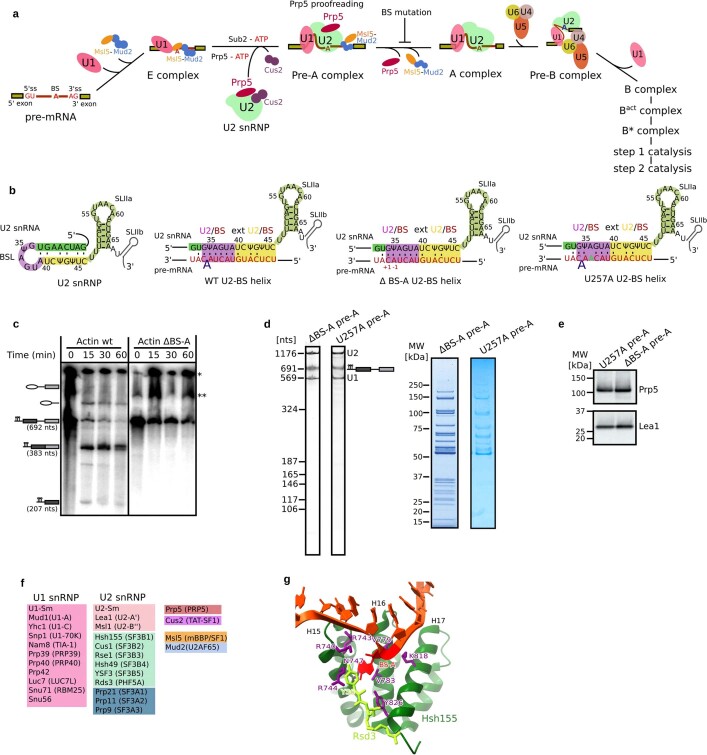
Biochemical characterization of *S. cerevisiae* pre-A spliceosomal complexes. **a**, Early assembly of the *S. cerevisiae* spliceosome. Whereas Prp5 and Tat-SF1 (Cus2 in yeast) are stable components of the human 17S U2 snRNP, they appear to be less-stably associated with the yeast U2 snRNP. The spliceosome undergoes numerous structural and compositional rearrangements during its assembly and catalysis of pre-mRNA splicing^34,35^. Conserved DEXH/D-box RNA helicases are important driving forces for these rearrangements, and also ensure the proper recognition of the branch site (BS) and the 5′- and 3′-splice sites (ss) via proofreading mechanisms^36,37^. Initially an E complex is formed in an ATP-independent manner. In the yeast E complex (also denoted the commitment complex), the 5′-ss is bound by U1 snRNP, and the BS and 3′-end of the intron are bound by a heterodimer of Msl5 and Mud2. RNP rearrangements that lead to the stable association of U2 snRNP and enable the formation of a U2–BS helix—in which an adenosine is bulged, specifying it as the nucleophile for catalytic step 1 of splicing—require the ATP-dependent action of the DEAD-box RNA helicases Sub2 (refs. ^38,39^; UAP56 in humans) and Prp5 (refs. ^1–4,40^). U2 nucleotides that base pair with the BS are initially sequestered in a stem-loop structure denoted the BSL^11,14^. Sub2 may free the BS region by displacing Msl5 (refs. ^41,42^), while Prp5 has been proposed to displace U2 snRNP proteins, including Cus2 (TAT–SF1 in humans), from the BSL^11,14^. This frees U2 nucleotides to base pair with the BS, and leads to the formation of the A complex with stably bound U2 snRNP. **b**, Structure of the BSL and U2–BS helices formed on an *Act* pre-mRNA wild-type (WT) BS (UACUA(**A**)C, where the BS-A is in bold), ΔBS-A (UACUAC**)** or U257A (UACAA(**A**)C) branch site. Note that the exact conformation of the U257A U2–BS helix is not clear. The U2–BS helix is highlighted in purple, and the extended U2–BS helix, in which the number and nature of base-pairing interactions varies depending on the pre-mRNA intron sequence, is highlighted in yellow. **c**, Deletion of the BS adenosine from the *Act* pre-mRNA stalls splicing before the first catalytic step. Splicing was performed in two independent experiments in yeast extract for 30 min at 23 °C with wild-type (lane 1) or ΔBS-A (lane 2) *Act* pre-mRNA containing MS2 aptamers for affinity purification. *Position of the loading well. **Band artefact not related to pre-mRNA splicing. For gel source data, see Supplementary Fig. 1. **d**, RNA (left gels) and protein (right gels) composition of purified yeast pre-A complexes formed on ΔBS-A and U257A *Act* pre-mRNA. RNA and protein were analysed on NuPAGE gels and visualized by staining with SYBR Gold or Coomassie, respectively, in two independent experiments. Note that fewer picomoles of the U257A pre-A complex were loaded onto the gel and, as a consequence, proteins of lower molecular weight are poorly or not at all visible. **e**, Prp5 is present in both U257A and ΔBS-A pre-A complexes. Proteins from affinity-purified U257A or ΔBS-A pre-A complexes (as indicated above each lane) were analysed by western blotting in two independent experiments with antibodies against *S. cerevisiae* Prp5 or Lea1 (used to ensure equal loading). **f**, Proteins localized in the *S. cerevisiae* pre-A complex and their human homologues (shown in parentheses). Only U1–70K, U1–A and U1–C have been identified as stable components of human U1 snRNPs. Human homologues of Snu56 and Prp42 have not been identified. **g**, Residues forming the BS-A-binding pocket. The bulged BS-A is bound in a pocket composed of residues R744, N747, V783 and Y826 of Hsh155 and residue Y36 of Rds3 (refs. ^7,8,43^). The BS-A ribose and 5′-phosphate are also located near Hsh155 residues K740 and K818, respectively. Most of these residues are evolutionarily highly conserved. A Hsh155 K818A mutation is lethal, as are mutations in residues of Hsh155 that contact the backbone of nucleotides directly adjacent to the bulged BS-A^19^. However, many of the Hsh155 residues forming the BS-A binding pocket are nonessential. That is, single alanine substitutions at K740, R744, N747 and V783 do not affect yeast viability, but they do affect recognition of the branch site^19^. Substitutions with bulkier amino acids decrease the use of nonconsensus branch sites, whereas substitutions with smaller amino acids increase usage^19^.

**Extended Data Fig. 2:**
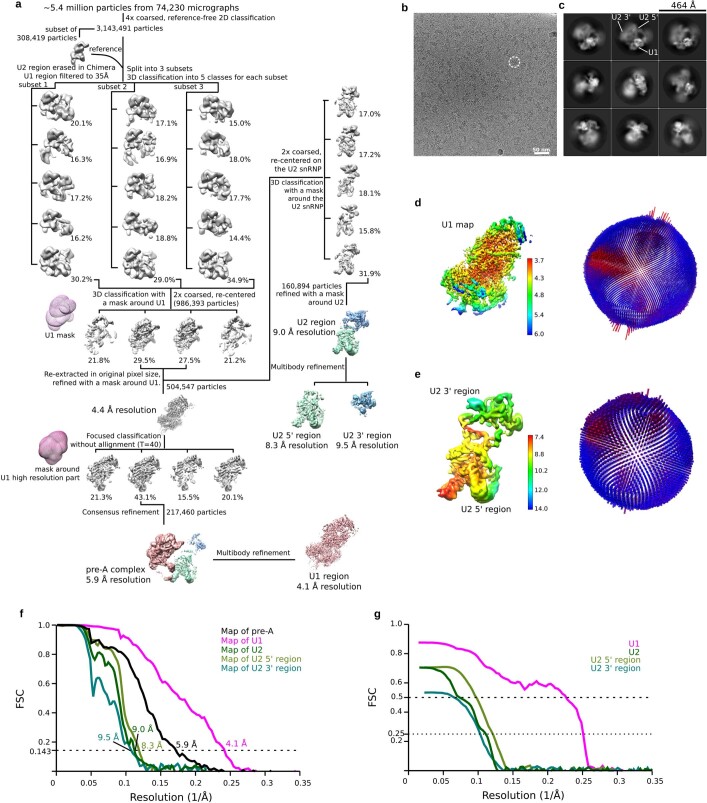
Cryo-EM and image processing for the ΔBS-A pre-A complex. **a**, Computation sorting scheme. All major image-processing steps are depicted. For a more detailed explanation, see the Methods section on ‘Image processing’. **b**, Typical cryo-EM micrograph (out of a total of 74,230) of the *S. cerevisiae* pre-A complex recorded at ×120,700 magnification with a Titan Krios microscope using a Falcon III direct electron detector operating in integration mode at a calibrated pixel size of 1.16 Å. **c**, Representative cryo-EM 2D class averages of the yeast pre-A complex reveal considerable flexibility between the U1 snRNP and the U2 snRNP. **d**, Left, local-resolution estimations of the cryo-EM reconstruction of the U1 snRNP. Right, plot showing the distribution of orientations for the particles contributing to the U1 reconstruction. **e**, Left, local-resolution estimations of the cryo-EM reconstructions of the U2 5′- and 3′-regions. Right, plot showing the distribution of orientations for the particles contributing to the reconstructions of the U2 5′- and 3′-regions. **f**, A Fourier shell correlation (FSC), calculated using the ‘post-processing’ routine in RELION, indicates a global resolution of 5.9 Å for the entire yeast pre-A complex and of 4.1 Å, 8.3 Å and 9.5 Å for the multibody-refined U1, U2 5′- and U2 3′-regions, respectively. The resolution was limited to 5.9 Å on average, owing to the movement of the U1 and U2 snRNPs relative to each other. Signal subtraction combined with local refinement improved the more stable U1 snRNP part to 4.1 Å resolution, whereas the bipartite U2 snRNP exhibited considerable internal flexibility and was refined only to 9 Å resolution. As the U2 5′-region—which is composed of the SF3b proteins, U2 SLIIa/b and the U2–BS helix—is attached to the more stable U1 snRNP and is therefore less flexible, further local classification and refinement improved the resolution of the U2 5′-region to 8.3 Å. The U2 3′-region—which comprises the U2 Sm core, U2 SLII, and Lea1 and U2-B′′ bound to U2 SLIV, plus the adjacent SF3a core (that is, regions of Prp9, Prp11 and Prp21 whose crystal structure has been determined previously^44^)—does not contact the U1 snRNP and therefore exhibits the greatest flexibility relative to the rest of the complex. **g**, Map versus model FSC curves for the U1, U2, U2 5′- and U2 3′-regions, using PHENIX mtriage^45^.

To isolate a spliceosome assembly intermediate formed directly before the A complex that still contains Prp5 (Extended Data Fig. 1a), we carried out splicing in *S. cerevisiae* cell extracts with an actin (*Act*) pre-mRNA in which the BS-A is deleted (Extended Data Fig. 1b). With this ΔBS-A substrate, splicing is blocked before catalytic step 1 (Extended Data Fig. 1c), consistent with previous results^6^. Spliceosomal complexes formed on ΔBS-A *Act* pre-mRNA lack the U4/U6.U5 tri-snRNP, but contain stoichiometric amounts of U1 and U2 snRNPs (Extended Data Fig. 1d). The proteins Prp5, Msl5 and Mud2 are also abundant, whereas Cus2 is absent (Extended Data Fig. 1e and Supplementary Table 1), indicating that these complexes stall after Prp5 hydrolyses ATP, but before the tri-snRNP has docked. We next carried out single-particle cryo-electron microscopy (cryo-EM) and determined the structure of the ΔBS-A complex at an average resolution of 5.9 Å (ranging from roughly 4.5 Å for U1 to approximately 15 Å for U2) (Extended Data Table 1 and Extended Data Fig. 2). Further classification and multibody refinement improved the resolution of the stable U1 snRNP region and adjacent U2 5′ region to 4.1 Å and 8.3 Å, respectively (Extended Data Fig. 2). By fitting known X-ray structures of spliceosome components into the EM density map (Extended Data Table 2), together with protein crosslinking coupled with mass spectrometry (CXMS) (Supplementary Table 2), we generated a three-dimensional (3D) model of the ΔBS-A complex (Fig. 1). This complex consists of two major elongated domains—comprising the U1 and bipartite U2 snRNPs—that are connected by two main bridges (Fig. 1).

## The U2–BS helix is not clamped by Hsh155

**Fig. 2:**
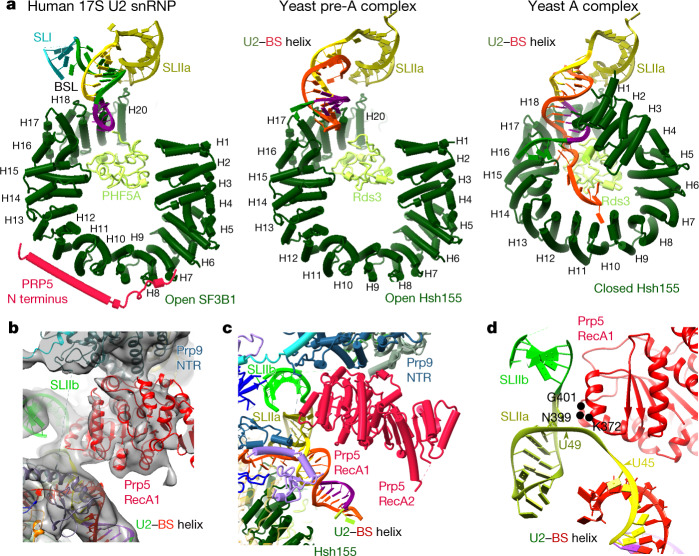
The Hsh155 HEAT domain has an open conformation in the pre-A complex. **a**, Conformation of the SF3B1 and Hsh155 HEAT domains and position of the U2–BS helix and U2 snRNA SLI and SLIIa in human 17S U2 snRNP (Protein DataBank (PDB) (https://www.rcsb.org) accession number 6Y5Q) and in the *S. cerevisiae* pre-A and A complexes (PDB 6G90). These domains were aligned via Hsh155 heat repeats 19–20, Rse1^BPA^ and U2 SLIIa. Olive green, SLIIa nucleotides; reddish orange, pre-mRNA branch-site nucleotides; purple, BSL nucleotides that later form the U2–BS helix; yellow, BSL nucleotides forming the extended part of the U2–BS helix; dark green, remaining BSL nucleotides; blue, SLI. **b**, Fit of Prp5^RecA1^ into the pre-A EM density. **c**, Location of the Prp5 RecA1 and RecA2 domains in the pre-A complex. **d**, Prp5^RecA1^ contacts U2 snRNA nucleotides that connect the U2–BS helix to U2 SLIIa. The positions of Prp5 amino acids (located outside of the SAT motif) that when mutated suppress branch-site mutations are indicated in black.

**Extended Data Fig. 3:**
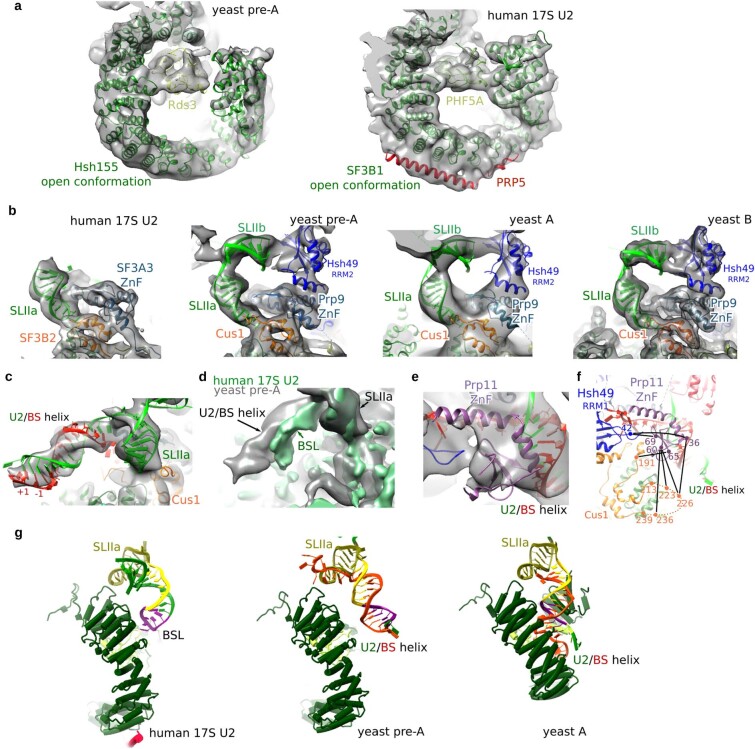
Open conformation of Hsh155^HEAT^ and spatial organization of the U2–BS helix and U2 SLIIa/b in the pre-A complex. **a**, Left, fit of Hsh155^HEAT^ and Rds3 to the pre-A EM density; and right, fit of SF3B1, PHF5A and the N-terminal helix of Prp5 to the human 17S U2 density (PDB accession code 6Y5Q). Previous biochemical studies showed that the N-terminal region (NTR) of yeast Prp5 binds to HEAT repeats (HRs) 1–6 and HR 9–12 of Hsh155 (ref. ^17^), and in the human 17S U2 snRNP cryo-EM structure, binding to SF3B1 HR 9–12 involves a long α-helix of the human Prp5^NTR^ (ref. ^11^). EM density that would accommodate an analogous α-helix of the yeast Prp5^NTR^ is not apparent in the pre-A complex. However, CXMS indicates that the Prp5^NTR^ still interacts extensively with Hsh155, albeit solely with HR 1–7 (see Extended Data Fig. 4b). Base pairing of U2 snRNA with the pre-mRNA branch site (BS) is stabilized by the major scaffolding protein SF3B1 (Hsh155 in *S. cerevisiae*) of the SF3b heteromeric complex, whose HEAT domain undergoes a conformational change during early spliceosome assembly. Hsh155^HEAT^ and SF3B1^HEAT^ exhibit an open conformation in the pre-A complex and the 17S U2 snRNP, respectively. The closed conformations of the HEAT domains of human SF3B1 and of yeast Hsh155 observed in the A to B^act^ spliceosomal complexes are very similar. Likewise, the open conformation, which we observe here for the first time for Hsh155, also appears to be highly similar in human U2 snRNP and the *S. cerevisiae* pre-A complex. **b**, Similar spatial organization of U2 snRNA SLIIa, Prp9^ZnF^ (SF3A3 in humans) and Cus1 (SF3B2 in humans) in the human 17S U2 snRNP (PDB 6Y5Q) and *S. cerevisiae* pre-A, A (PDB 6G90) and B (PDB 5NRL) complexes. Aligned via U2 SLIIa and HR 19–20 of SF3B1/Hsh155. In the pre-A complex, SLIIb can be localized downstream of SLIIa and is bound by RRM2 of Hsh49. **c**, Fit of a modelled 13-base-pair extended U2–BS helix, lacking a bulged A, to EM density adjacent to SLIIa in the pre-A complex. **d**, Overlay of EM density accommodating the U2–BS helix in the yeast pre-A complex (grey) and EM density accommodating the BSL in human 17S U2 (green) (PDB 6Y5Q). Aligned via U2 SLIIa and HR 19–20 of SF3B1/Hsh155. The sequences of the *S. cerevisiae* and human U2 BSLs are highly conserved^14^, allowing a meaningful comparison with the fit of the yeast U2–BS helix. Although the BSL in yeast and human is predicted to form a 9-base-pair stem^14^, in the human 17S U2 snRNP, the base of the BSL stem is contacted by a short helix of SF3A3 (designated the separator helix), which ensures that the stem is only 8 base pairs in length^11^. **e**, Fit in the pre-A EM density of the Prp11^ZnF^ at the top of the U2–BS helix. **f**, Protein crosslinks supporting the positioning of the Prp11^ZnF^ in the pre-A complex. Numbers (colour coded to match protein colours) indicate the positions of crosslinked lysine residues, which are connected by black lines. The Prp11^ZnF^ appears to act independently of the other SF3a proteins and to dock to the end of the extended U2–BS helix concomitantly with, or soon after, its formation. As the Prp11^ZnF^ has thus far been observed at this position solely after formation of the U2–BS helix, its location in the pre-A complex is consistent with the conclusion that a U2–BS helix has formed. It is likely that the Prp11^ZnF^ and the Prp9 separator helix may cooperate in keeping additional intron nucleotides from interacting with the U2 snRNA, and at the same time in stabilizing the end of the U2–BS helix. During clamping of the U2–BS helix by Hsh155^HEAT^, Prp11^ZnF^ moves together with the U2–BS helix and remains associated with the end of the helix in the A, pre-B, B and B^act^ complexes. **g**, Side view showing that the U2–BS helix is located further away from the C-terminal HRs of Hsh155^HEAT^ in the pre-A complex compared with its position in the *S. cerevisiae* A complex and the position of the BSL in human 17S U2 snRNP. Aligned via Hsh155 HR 19–20, Rse1^BPA^ and U2 SLIIa. Olive green, SLIIa nucleotides; red orange, pre-mRNA BS nucleotides; purple, BSL nucleotides that later form the U2–BS helix; yellow, BSL nucleotides forming the extended part of the U2–BS helix; dark green, the remaining BSL nucleotides; blue, SLI. Movement away from Hsh155^HEAT^ would be needed to free the SLI-containing 5′-end of U2 snRNA to undergo the rotational movements necessary to generate an extended U2–BS helix. During the transition from the pre-A to the A complex, the U2–BS helix moves back towards the Hsh155 C-terminal HRs such that the corresponding region of the U2 snRNA that contacts the C-terminal HRs in 17S U2 is located in a similar position in the A complex.

Hsh155^HEAT^ adopts a closed conformation after U2 has integrated stably into the spliceosome, clamping the U2–BS helix and binding the bulged BS-A in a pocket formed by Rds3 (PHF5A in humans; see Extended Data Fig. 1f for a summary of yeast and human protein names) and HEAT repeats 15–17 of Hsh155 (refs.^7–10^) (Extended Data Fig. 1g). It is unclear at present what triggers this functionally important structural change. In the ΔBS-A complex, Hsh155^HEAT^ exhibits an open conformation, as in the isolated human 17S U2 (ref. ^11^) but in notable contrast to its conformation in yeast A complexes (Figs. 1, 2a and Extended Data Fig. 3a) and pre-B, B and B^act^ complexes^7,8,12,13^. Stem–loop (SL) IIa of U2 snRNA is bound by Hsh155, Cus1 and Prp9 in the ΔBS-A complex, in a similar manner to that seen in the human 17S U2 snRNP and subsequently formed spliceosomal complexes (Extended Data Fig. 3b). An extended helical density element is located directly upstream of SLIIa. This element is longer than the U2 branchpoint-interacting stem-loop (BSL), which is found in the isolated U2 snRNP and sequesters U2 nucleotides that base pair with the branch site^11,14^ (Extended Data Fig. 3c, d). A modelled, extended U2–BS helix—lacking a bulged BS-A and comprising 13 base pairs—fits well into this density element (Extended Data Fig. 3c), indicating that an extended U2–BS helix has formed. On the basis of CXMS data, the Prp11 zinc finger (Prp11^ZnF^) could be positioned at the top of the U2–BS helix (Extended Data Fig. 3e, f), akin to its position in A, pre-B, B and B^act^ complexes^7,8,12,13,15^. In the ΔBS-A complex, the U2–BS helix is located further away from the carboxy (C)-terminal HEAT repeats of Hsh155^HEAT^ compared with its position in the A to B^act^ complexes, and it is not sequestered by Hsh155^HEAT^ (Fig. 2a and Extended Data Fig. 3g). Thus, formation of the U2–BS helix alone does not appear to trigger closure of Hsh155^HEAT^. As the U2–BS helix has formed, but Hsh155^HEAT^ still exhibits an open conformation and Prp5 is stably bound (see below), we conclude that ΔBS-A complexes are stalled at a pre-A-complex stage, after Prp5-mediated formation of the U2–BS helix, but during/before it carries out its proofreading function.

## Location of Prp5 in the pre-A complex

**Extended Data Fig. 4:**
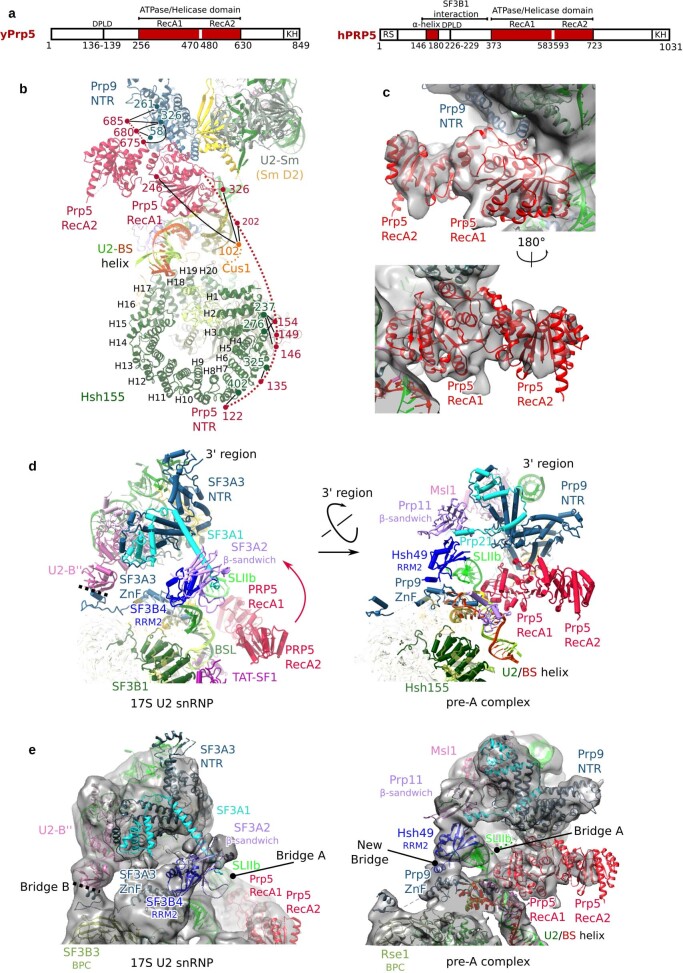
Repositioning of the Prp5 RecA domains and the U2 3′-region during formation of the pre-A complex. **a**, Domain organization of the *S. cerevisiae* (y) and human (h) DEAD-box helicase Prp5, with the amino-acid boundaries of each domain indicated below. **b**, Protein crosslinks support the positions of the Prp5 NTR and RecA domains in the pre-A complex. Numbers (colour coded to match protein colours) indicate the positions of crosslinked lysine residues, which are connected by black lines. The proposed path of Prp5 amino acids located more N-terminally of the RecA domains is indicated by a dashed line. That the Prp5 NTR and RecA1 domains, but not RecA2 (and presumably also its C-terminal region), interact with other pre-A components is consistent with previous studies showing that, after destabilization of the U2 BSL, Prp5^NTR^ and Prp5^RecA1^ are sufficient for the subsequent ATP-independent function of Prp5 during A-complex formation^14^. **c**, Two different views of the fit of the Prp5 RecA1 and RecA2 domains in an open conformation into the pre-A EM density (low-pass filtered to 10 Å). A closed conformation of the Prp5^RecA^ domain does not fit well to the EM density (not shown). The open conformation of Prp5 found in the pre-A complex indicates that, after ATP hydrolysis, the RecA domains are able to transit spontaneously from the closed conformation back to the open conformation while probably remaining bound to U2. **d**, **e**, The positions of the Prp5^RecA^ domains and the U2 3′-region plus SF3a proteins, relative to SF3b, are different in the human 17S U2 snRNP and the yeast pre-A complex. Aligned via U2 SLIIa and HR 19–20 of SF3B1/Hsh155. A cryo-EM structure of an isolated *S. cerevisiae* U2 snRNP is currently lacking. However, the high conservation of the sequence of yeast U2 proteins and their human homologues, and the similar structures of their conserved domains, suggests that the molecular architecture of the isolated U2 snRNP is similar in *S. cerevisiae* and humans. Thus, a comparison of the structures of the human 17S U2 snRNP and yeast pre-A complex reveals structural remodelling that the U2 snRNP most likely undergoes during formation of the pre-A complex. An alignment of the U2 5′-region in both complexes suggests that the U2 3′-region is repositioned after U2 stably interacts during formation of the pre-A complex. Specifically, the U2 3′-domain (that is, the 3′-region minus the SF3a core) and the Prp9^NTR^ rotate towards the Prp5^RecA^ domains, whereas the Prp11 β-sandwich and Hsh49^RRM2^ move towards Prp9^ZnF^. The shifted position of the U2 3′-region is stabilized by different molecular bridges formed between the U2 3′- and 5′-regions. In the pre-A complex, the bridge formed by U2-B′′ RRM2, Prp9 (human SF3A3) and Rse1^BPC^ (human SF3B3) in the 17S U2 snRNP (denoted bridge B) is disrupted, which allows the 3′-region to move further away from the Rse1^BPC^. This then allows Hsh49^RRM2^ to dock on top of the Prp9^ZnF^, and by binding to Prp9 on one side and the Prp11 β-sandwich domain on the other, a new bridge involving Hsh49^RRM2^ is formed. Moreover, in the isolated human 17S U2 snRNP, U2 SLIIb forms a second bridge (denoted bridge A) between the U2 3′- and 5′-regions that is not stabilized and is only poorly resolved. By contrast, in the pre-A complex, Hsh49^RRM2^ now binds to the loop of SLIIb and thereby stabilizes the position of SLIIb.

Prp5 was initially proposed to ‘proofread’ formation of the U2–BS helix by coordinating the rate of U2–BS base pairing with its ATPase activity^5^. However, more recent studies uncovered a correlation between increased Prp5 retention in early spliceosomal complexes and decreased tri-snRNP recruitment, suggesting that the physical presence of Prp5, rather than its ATPase activity, has a key role in its proofreading function^16^. In the human 17S U2 snRNP, the Prp5 RecA (Prp5^RecA^) domains sequester the BSL together with the TAT–SF1 protein and the C-terminal HEAT repeats of SF3B1 (ref. ^11^), thereby preventing formation of the U2–BS helix. We find here that, in the yeast pre-A complex, Cus2 (human TAT–SF1) has dissociated and the Prp5^RecA^ domains are located between the U2 3′-region and the U2–BS helix (Fig. 2b, c and Extended Data Fig. 4a–d). Compared with their position in 17S U2 snRNP, the Prp5^RecA^ domains in the pre-A complex are located further away from Hsh155^HEAT^, with RecA1 fitting well into the niche formed by the amino (N)-terminal region of Prp9 (Prp9^NTR^), with which it interacts, and the U2–BS helix (Fig. 2b, c and Extended Data Fig. 4c–e). Moreover, Prp5^RecA1^ now contacts the U2 snRNA strand that connects SLIIa and the U2–BS helix (Fig. 2d). This is consistent with the crosslinks of Prp5 to this region (nucleotides 45–49) of yeast U2 snRNA observed previously with a Prp5-associated *S. cerevisiae* spliceosomal complex (designated the Prp5-associated intermediate complex, or FIC) formed on a pre-mRNA with a mutated branch site^16^. Finally, CXMS indicates that the NTR of Prp5 interacts extensively with Hsh155 HEAT repeats 1–7 in the pre-A complex (Extended Data Fig. 4a, b), consistent with previous biochemical studies^17^.

## Pre-A formation involves U2 remodelling

Comparison of the structures of the yeast pre-A complex and human 17S U2 snRNP suggests that, in addition to Prp5^RecA^, there are other changes in the organization of U2 components during pre-A formation. Relative to its position in 17S U2, the U2 3′-region has moved (Extended Data Fig. 4d, e) and its new location in the pre-A complex is stabilized by newly formed contacts between the U2 3′- and 5′-regions (Extended Data Fig. 4d, e). This movement is a prerequisite for formation of the pre-A complex, as it generates the binding pocket for the repositioned Prp5^RecA1^ (Fig. 2b, c and Extended Data Fig. 4). In the pre-A complex, Prp5^RecA1^ establishes new contacts with the shifted 3′-region by interacting with Prp9^NTR^ (Fig. 2b, c and Extended Data Fig. 4), preventing the further movement of the U2 3′-region that is ultimately required to allow the tri-snRNP to dock to the A complex (see below).

## Prp40 bridges U1 and U2 snRNP in pre-A complex

**Extended Data Fig. 5:**
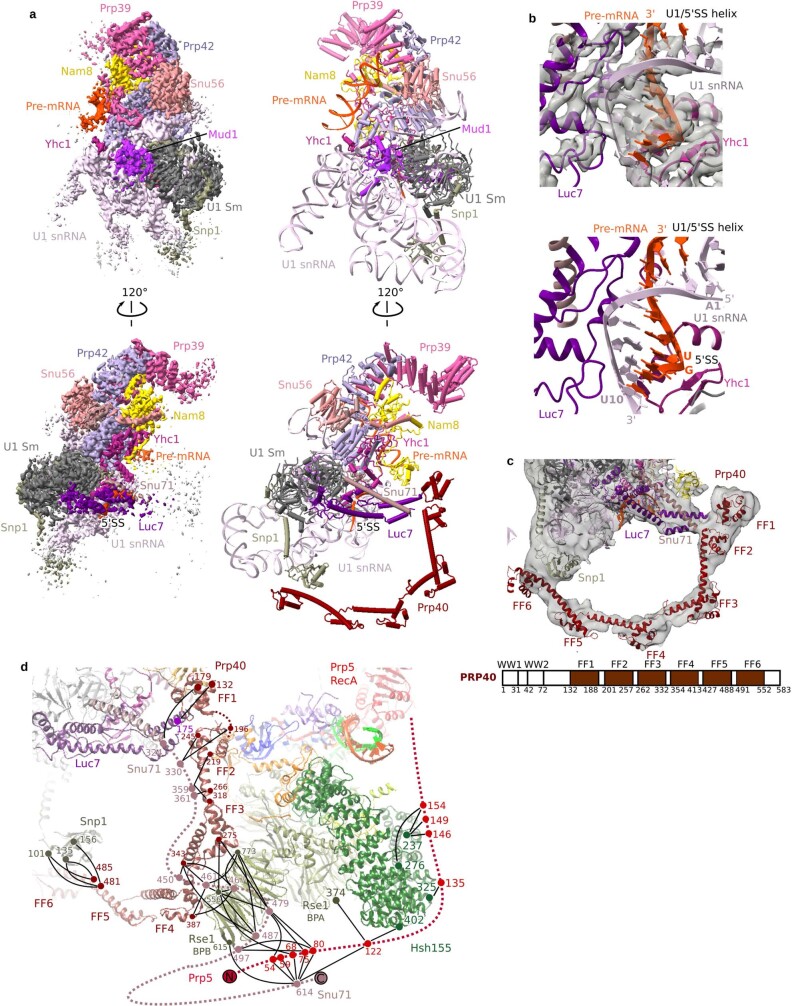
Molecular architecture of the U1 snRNP in the pre-A complex. **a**, Two different views of the spatial organization of the yeast U1 snRNP, with the density shown on the left and the molecular model on the right. **b**, The U1–5′ss helix is stabilized in the pre-A complex by Luc-7 and Yhc1, in the same manner as in the yeast E and A complexes. Top, fit of the U1–5′ss helix plus Luc7 and Yhc1 to the pre-A EM density. Bottom, the U1–5′ss helix and adjacent proteins. **c**, Fit of the Prp40 FF1–6 domains in the pre-A EM density. Top, domain organization of the *S. cerevisiae* Prp40 protein; below, amino-acid boundaries of each domain. WW, domain containing two conserved tryptophans that are spaced 20–22 amino acids apart; FF, domain containing two conserved phenylalanines at its N and C termini. **d**, Protein crosslinks between Prp40, Prp5^NTR^ and other pre-A-complex proteins. Numbers (colour coded to match protein colours) indicate the positions of crosslinked lysine residues, which are connected by black lines. Prp40, Snu71 and Luc7 form a stable trimer^46^ that in the cryo-EM structure of the yeast E complex^18^ bridges the U1 snRNP to the branch site, and we show here that they also help to bridge U1 snRNP to U2 during the early stages of prespliceosome formation. In humans and in *Schizosaccharomyces pombe*, Prp5 facilitates formation of the A complex by bridging the U1 and U2 snRNPs^4^, with the Prp5 N-terminal RS domain interacting with proteins of the SF3b complex^21^. Although *S. cerevisiae* Prp5 lacks an N-terminal RS domain, CXMS data indicate that its N terminus also interacts with Snu71 and Rse1^BPB^. Therefore, the bridge formed by Rse1 and the Prp40–Luc7–Snu71 trimer in the *S. cerevisiae* pre-A complex probably serves as an anchoring point for Prp5’s N terminus.

**Extended Data Fig. 6:**
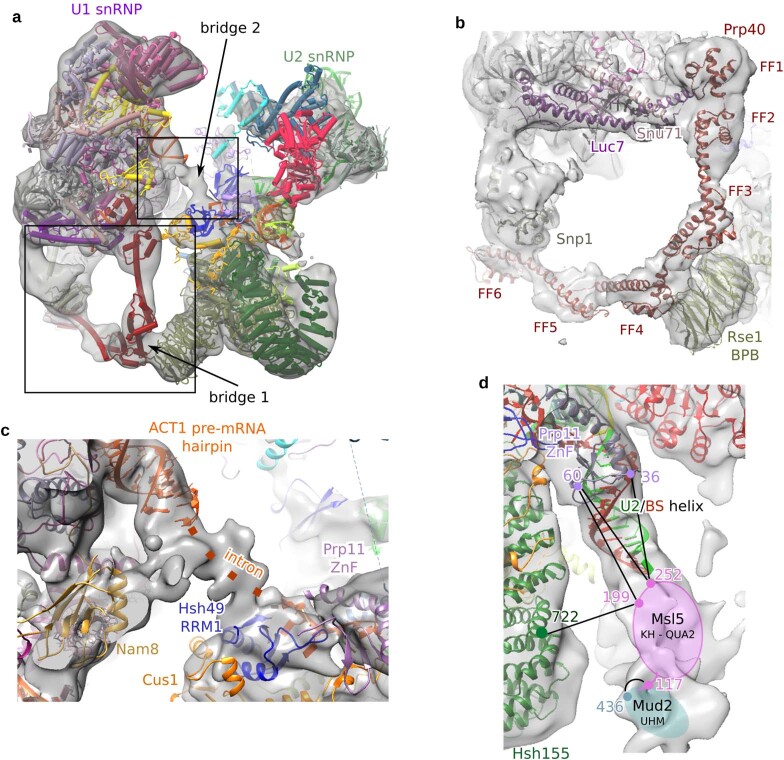
Molecular bridges connecting the U1 and U2 snRNPs in the pre-A complex. **a**, Fit of the molecular model of the entire pre-A complex into the EM density (low-pass filtered). The two main bridges that connect the U1 and U2 snRNPs are indicated by arrows. The boxes indicate the regions expanded in **b**, **c**. **b**, Close-up of bridge 1 formed mainly by the interaction of Prp40 with Rse1. Bridge 1 is disrupted during the transition from the pre-A to the A complex (see Fig. 3 and Supplementary Video 1). Deletion analyses of Prp40 showed that although FF domains 3–6 are dispensable for yeast viability, they convey a considerable growth disadvantage when absent^46^. There is also evolutionary conservation of the presence of four or more of the FF domains in Prp40 from various organisms^46^, suggesting that FF3 and FF4 have important roles during spliceosome assembly and/or splicing. Our crosslinking data indicate that Snu71 also extensively contacts Rse1^BPB^ and FF2 of Prp40 (see Extended Data Fig. 5d). It is conceivable that, in the absence of Prp40 FF3–FF4, Snu71 still interacts with Rse1^BPB^, the latter being a protein–protein interaction domain that interacts with different proteins in the subsequently formed B and B^act^ spliceosomal complexes. **c**, Close up of bridge 2 that is formed by intron nucleotides between the U2–BS helix and the 5′-ss. By analogy to the situation in later spliceosomal complexes^12^, these intron nucleotides are likely to be chaperoned by Hsh49^RRM1^ and Prp11^ZnF^. Intron nucleotides of the *Act* pre-mRNA (but not of other pre-mRNAs such as *Ubc4*) form a hairpin that can be localized adjacent to the U1 snRNP already in the E complex^18^. The resolution is not sufficient to determine the exact intron nucleotides comprising this stem. Note that the intron hairpin is not part of bridge 2. **d**, A lower threshold reveals EM density below the U2–BS helix, adjacent to the open Hsh155^HEAT^ domain, that probably corresponds to the Mud2–Msl5 dimer. The EM map is low-pass filtered to 30 Å resolution. Protein crosslinks supporting the localization of Msl5–Mud2 adjacent to the U2–BS helix are shown. Numbers (colour coded to match protein colours) indicate the positions of crosslinked lysine residues, which are connected by black lines. Msl5–Mud2 could not be precisely modelled into the EM density, presumably because of their structural flexibility. However, on the basis of CXMS data, we tentatively position Msl5–Mud2 into weak density directly downstream of the branch site, close to the U2–BS helix, with Mud2 being bound to the 3′-end of the intron. Formation of the U2–BS helix requires that Msl5 hands the branch site over to the U2 snRNA, and thus Msl5 should already be displaced from the branch site in the pre-A complex. Therefore, retention of Msl5–Mud2 close to the U2–BS helix would be consistent with the binding of Mud2 to the intron downstream of the branch site.

The U1 snRNP structure in the pre-A complex (Extended Data Fig. 5a) is highly similar to that observed in the yeast E, A and pre-B complexes^13,15,18^, indicating that U1 does not undergo substantial remodelling during early spliceosome assembly. As in the aforementioned complexes, base pairing between the 5′-splice site and the U1 snRNA is also stabilized by Yhc1 and Luc7 in the pre-A complex (Extended Data Fig. 5b). Although only three FF domains of Prp40 (domains 4–6) could be located in the yeast E complex^18^, CXMS allowed us to map all six of the FF domains of Prp40 in the pre-A complex (Fig. 1 and Extended Data Fig. 5c, d). FF1 and FF6 bind Luc7 and Snp1, respectively, tethering Prp40 to U1, while FF2–FF5 form an extended binding platform that interacts with numerous proteins, including Snu71 and U2 Rse1. The interaction of FF4 with the WD40 β-propeller domain B (BPB) of Rse1 forms a bridge between the U1 and U2 snRNPs (denoted bridge 1) (Extended Data Figs. 5d, 6a, b) that is not observed in yeast A complexes^15^. Bridge 1 also contains the C-terminal region of Snu71, as numerous crosslinks between it and the FF2 and FF3 domains of Prp40, as well as with Rse1^BPB^, are detected (Extended Data Fig. 5d). In the pre-A complex, U1 and U2 are connected by a second bridge comprising intron nucleotides upstream of the branch site (Extended Data Fig. 6a, c). Although the Prp40 WW domain and Msl5–Mud2 could not be localized based solely on the EM density, CXMS indicates that Msl5–Mud2 is likely to be located near the U2–BS in the pre-A complex, and furthermore remains bound to the Prp40 WW domain (Extended Data Fig. 6d and Supplementary Table 2).

## Pre-A complex with a U257A branch-site mutation

**Extended Data Fig. 7:**
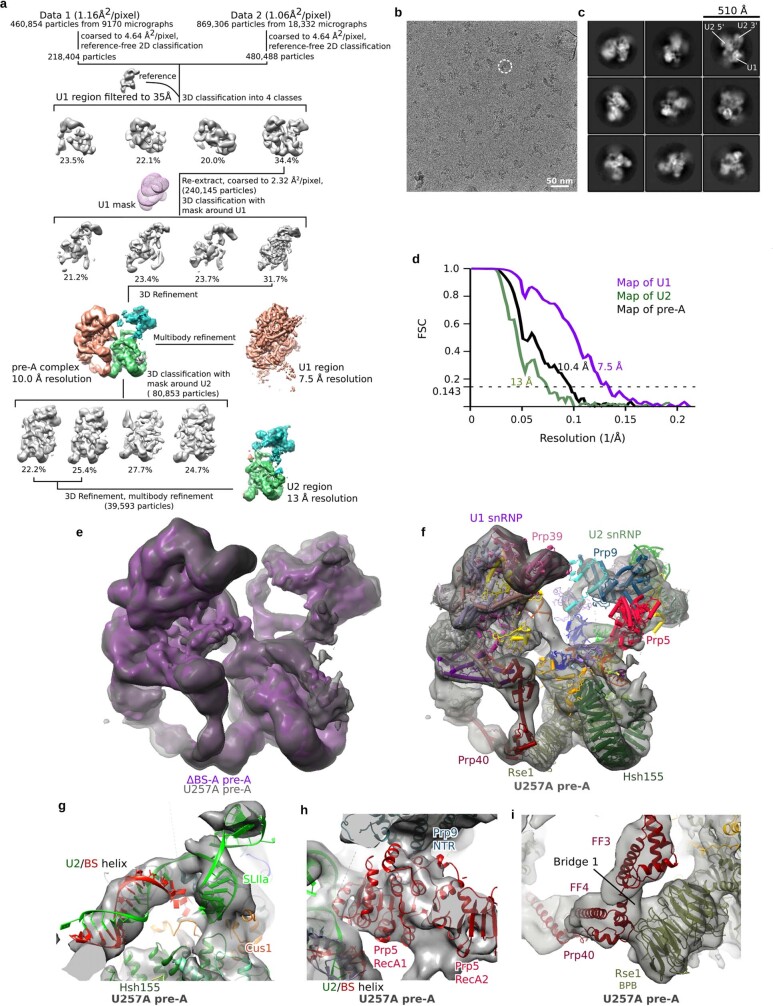
Cryo-EM and image-processing of the U257A pre-A complex. **a**, Computation sorting scheme, with all major image-processing steps depicted. For a more detailed explanation, see the Methods section on ‘Image processing’. **b**, Typical cryo-EM micrograph (out of a total of 27,502) of the *S. cerevisiae* U257A pre-A complex recorded at ×120,700 magnification with a Titan Krios microscope using a Falcon III direct electron detector operating in integration mode at a calibrated pixel size of 1.16 Å. **c**, Representative cryo-EM 2D class averages of the yeast U257A pre-A complex. **d**, FSC calculated using the ‘Post-processing’ routine in RELION indicates a global resolution of 10.4 Å for the entire yeast U257A pre-A complex, and resolutions of 7.5 Å and 13 Å for the multibody-refined U1 and U2 regions, respectively. The global resolution was lower than that of the ΔBS-A pre-A complex, mainly because of the lower number of particles analysed. **e**, Overlay of the EM densities of the ΔBS-A (purple) and U257A (grey) pre-A complexes. **f**, Fit of the 3D model of the ΔBS-A pre-A complex into the EM density of the U257A pre-A complex. Note that, for both complexes, density encompassing Prp5 is first observed at a lower threshold. An extended U2–BS helix has also formed in complexes formed on the U257A mutant. However, the precise conformation of the helix cannot be discerned. The Hsh155^HEAT^ domain is in an open conformation and Prp5 is still bound at the same position, and the same U1–U2 bridges are also observed, indicating that the U257A complexes are also stalled at the same pre-A stage. **g**, Fit of the extended U2–BS helix from the ΔBS-A pre-A complex into the U257A pre-A EM density. **h**, Fit of the Prp5^RecA^ domains and U2–BS helix from the ΔBS-A pre-A model into the EM density of the U257A pre-A complex. **i**, Fit of the Prp40 FF domains and Rse1^BPB^ (which comprise bridge 1) from the ΔBS-A pre-A model into the EM density of the U257A pre-A complex.

Mutations in the conserved yeast branch-site sequence upstream of the BS-A that weaken the U2–BS interaction—including a U-to-A mutation at position 257 of *Act* pre-mRNA (two nucleotides upstream of the BS-A) (Extended Data Fig. 1b)—do not completely block splicing but do lead to the accumulation of spliceosomes in which Prp5 is retained but the tri-snRNP has not yet joined^5,16^. To determine whether this mutation stalls spliceosome assembly at the pre-A stage, we purified the complexes that form on *Act* U257A pre-mRNA and determined their cryo-EM structure (Extended Data Fig. 7). The RNA and protein compositions of the purified U257A and ΔBS-A complexes were identical (Extended Data Fig. 1d, e and Supplementary Table 1), and an overlay of the U257A and ΔBS-A complexes revealed a highly similar, if not identical, structure at the present level of resolution (Extended Data Fig. 7). Indeed, the structural model of the ΔBS-A pre-A complex fits well without further adjustment into the EM density of the U257A complex (Extended Data Fig. 7). Thus, with the U257A mutant, an extended U2–BS helix has also formed, Hsh155^HEAT^ is in an open conformation, and Prp5^RecA1^ is docked to Prp9^NTR^ and situated close to the U2 snRNA, indicating that U257A complexes are also stalled at the same pre-A stage. The highly similar structure of both pre-A complexes indicates that they represent a physiologically relevant intermediate that—at least in the case of U257A—can also progress along the wild-type spliceosome assembly pathway.

## Dynamics of the pre-A to A transition

**Fig. 3:**
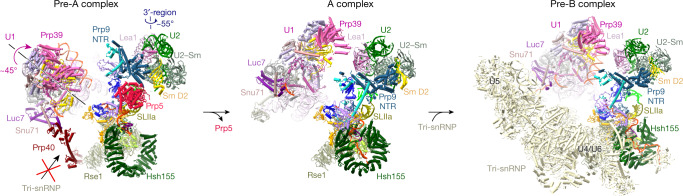
Prp5 blocks the repositioning of U1 and U2 snRNPs that is required to form the tri-snRNP-binding site. Molecular organization of U1 and U2 snRNPs in *S. cerevisiae* pre-A, A (PDB 6G90) and pre-B complexes (PDB 5ZWM and PDB 5ZWN). Movements of U1 and U2 snRNPs during the pre-A to A transition are indicated by curved arrows. All structures were aligned as in Fig. 2. For simplicity, the U1 snRNA stem-loops in the poorly resolved region of the U1 snRNP are not shown in the pre-A, A and pre-B complexes.

**Extended Data Fig. 8:**
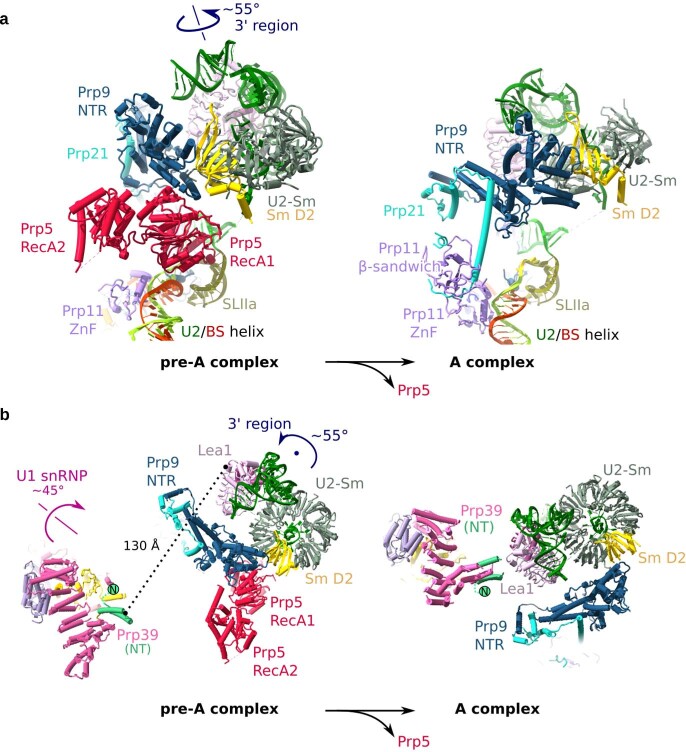
Movement of U1 and U2 snRNPs during the transition from the pre-A to the A complex. **a**, Close-up of the rotation of the U2 3′-region after the release of Prp5. The 3′-region rotates around the indicated axis by roughly 55°. To better show the movement of the 3′-region, the SmD2 protein is in yellow. For simplicity, only the 3′-region of U2 plus U2 SLII and the U2–BS helix are shown in the pre-A complex and the yeast A complex (PDB 6G90). The pre-A and A complexes are aligned via U2 SLIIa and HR 19–20 of Hsh155. **b**, Close-up of the movement of the U1 snRNP and 3′-region of U2. A top view is shown, with the black dot indicating the pivot point of the U2 3′-region, which rotates by roughly 55° in the plane of the paper. For simplicity, only the region of U1 snRNP that contains Prp39 is shown. The U1 snRNP rotates around the indicated axis by roughly 45°. In the pre-A complex, Prp39 and Lea1 are separated by roughly 130 Å, but the movements of U1 and U2 bring them into close proximity in the A complex. Even though Lea1 is not essential in *S. cerevisiae*, its depletion prevents formation of the A complex, and adding back Lea1 restores A-complex assembly^47^. The Prp39–Lea1 interaction is a structural marker for the formation of a mature A complex, and as such its absence in the pre-A complex is a clear indication that our complex has stalled at an earlier assembly stage. This interaction is also maintained in the pre-B complex^13^ and is therefore also a structural marker for the conformation that allows joining of the tri-snRNP.

Comparison of our pre-A complex with the previously published yeast A complex^15^ reveals that the transition from the pre-A to the A complex involves large-scale remodelling that requires displacement of Prp5 (Fig. 3, Extended Data Fig. 8 and Supplementary Video 1). First, the U2 3′-region rotates by roughly 55° relative to the U2 5′-region during A-complex formation. In the pre-A complex, this rotational movement is prohibited by the Prp5^RecA^ domains, which bind in a mutually exclusive manner with the new position of Prp9^NTR^, the long α-helix of Prp21 and the Prp11 β-sandwich, in the subsequently formed A complex. Second, U1 snRNP rotates by roughly 45° during the transition from the pre-A to the A complex, such that Prp39 now interacts with Lea1. A prerequisite for U1 movement is the dissociation of Prp40 from Rse1, and thus the apparent dissolution of U1–U2 bridge 1 (Fig. 3). The repositioning of U1 and the U2 3′-region is essential to generate the binding platform needed to dock the U4/U6.U5 tri-snRNP during formation of the pre-B complex (Fig. 3). Our studies thus provide a structural explanation for why the docking of tri-snRNP is inhibited when Prp5 is retained in yeast prespliceosomes^16^.

## Mechanism of proofreading by Prp5

**Fig. 4:**
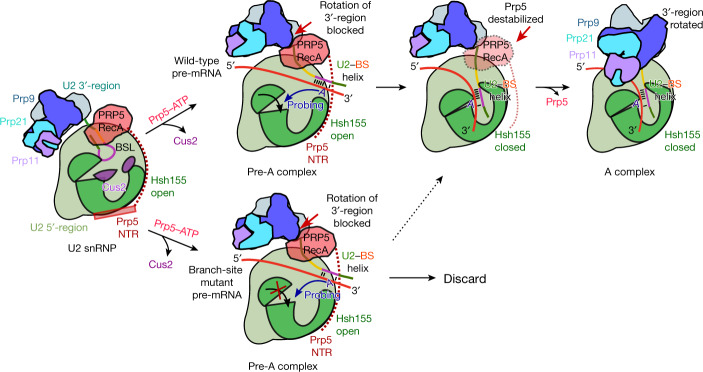
Model for Prp5-mediated proofreading of the U2–BS helix. For simplicity, in this depiction of our proposed mechanism of proofreading by Prp5, Rds3 of the BS-A-binding pocket is not shown, and the U1 snRNP has been omitted from the spliceosomal pre-A and A complexes. The closure of Hsh155^HEAT^ and release of Prp5 may be highly coordinated events that occur simultaneously rather than sequentially. The dashed arrow indicates that some mutations in the branch-site sequence, including U257A, do not completely abolish conversion of the pre-A complex into the A complex.

The cryo-EM structures presented here provide structural insights into the mechanism by which Prp5 proofreads the U2–BS helix (Fig. 4). The pre-A and 17S U2 structures are consistent with a model in which, after U2 interacts with the E complex, ATP hydrolysis by Prp5 leads to release of Cus2 and unwinding of the BSL. This allows formation of the U2–BS helix and repositioning of the U2 3′-region and Prp5^RecA^, generating the pre-A complex (Fig. 4). The new proofreading (or rather, ‘fidelity checkpoint’) position of Prp5^RecA^ in the pre-A complex transiently prevents the further movement of the U2 3′-domain needed to form an A complex that can subsequently bind the tri-snRNP. As deletion of the BS-A hinders the closure of Hsh155^HEAT^, but does not affect the stability of the U2–BS helix per se, correct binding of the bulged BS-A by Hsh155^HEAT^ and Rds3 is likely to be a major trigger for the conformational change in Hsh155^HEAT^. Furthermore, in the pre-A complex, the U2–BS helix is probably flexible, enabling it to intermittently move closer to Hsh155^HEAT^, which ‘probes’ for its presence. Thus, we propose that when a stable U2–BS with a bulged BS-A is formed, movement of the U2–BS into the open Hsh155^HEAT^ leads to insertion of the BS-A into its binding pocket and closure of the HEAT domain (Fig. 4). Previous mutational analyses of Hsh155 indicated that alignment of the U2–BS duplex with conserved, positively charged amino acids in the C-terminal half of Hsh155 is crucial for closure^19^, and this alignment could thus help to properly position the bulged BS-A in its binding pocket.

Closure of Hsh155^HEAT^ would destabilize not only the Prp5^NTR^ that binds to it, but also the Prp5^RecA^ domains (Fig. 4). Although the latter do not interact with Hsh155^HEAT^, the coordinated movement of Hsh155^HEAT^ and Prp5^NTR^ and the U2 snRNA nucleotides contacted by Prp5^RecA1^ could also lead to the displacement of Prp5^RecA^ and subsequent release of the entire Prp5 protein. Release of Prp5 would then allow rotation of the U2 3′-region and formation of an A complex (Fig. 4). If the U2–BS helix lacks a bulged BS-A, binding of the latter by Hsh155^HEAT^ and Rds3 would be blocked (Fig. 4). This would prevent the closure of Hsh155^HEAT^ and release of Prp5, blocking the progression of spliceosome assembly and potentially targeting the stalled complex for discard (Fig. 4). As branch-site mutations that destabilize the U2–BS duplex also hinder spliceosome assembly and lead to Prp5 retention^5,16^, the stability of the U2–BS duplex per se may also affect the conformational state of Hsh155^HEAT^ in pre-A complexes. A destabilized U2–BS helix could also potentially prevent proper bulging of the BS-A^20^, and in this way hinder closure of Hsh155^HEAT^.

Various mutations in Prp5 can suppress branch-site mutations, including those in the Prp5 DPLD motif (which is important for the interaction of Prp5 with the U2 snRNP^21^) and also mutations in its SAT motif and those in adjacent regions of RecA1 that have no effect on ATPase activity, such as K372E, N399D and G401E^5,21^. The Prp5^N399D^ mutant, the SAT mutant TAG and the DPLD mutant AAAA have reduced affinity for yeast prespliceosomes and enhance the binding of tri-snRNPs to spliceosomes^16^. Our pre-A structures provide insight into how some of these mutations may destabilize Prp5 binding. In pre-A complexes, the Prp5^NTR^ containing the DPLD motif still interacts with Hsh155^HEAT^ (Extended Data Fig. 4b), and thus mutation of this motif is likely to destabilize the Prp5^NTR^–Hsh155^HEAT^ interaction. In pre-A complexes, amino acids K372, N399 and G401 are located in the Prp5 region that interacts with the single-stranded U2 snRNA between SLIIa and the U2–BS helix (Fig. 2d). Consistent with less-stable Prp5 binding, these mutations introduce a negative charge that would destabilize the RecA1–U2 snRNA interaction. Although the SAT motif is not located at a Prp5–protein interface, some SAT mutations might alter the conformation of RecA1, thereby indirectly destabilizing its interaction with Prp9^NTR^. Indeed, several SAT mutations alter the equilibrium between the open and closed conformations of the Prp5^RecA^ domains^22^. Together, our results indicate that Prp5 does not proofread the U2–BS helix directly, but instead proofreads the RNP conformation of pre-A complexes, and hinders progression of spliceosome assembly if mutations in the branch site alter the formation of a productive, closed conformation of Hsh155^HEAT^.

## Methods

No statistical methods were used to predetermine sample size. The experiments were not randomized, and investigators were not blinded to allocation during experiments and outcome assessment.

### Preparation of yeast whole-cell extracts

Yeast whole-cell extracts were prepared from the *Saccharomyces cerevisiae* 3.2.AID/CRL2101 strain (*MAT*alpha, *prp2-1*, *ade2*, *his3*, *lys2-801*, *ura3*) (a gift from R.-J. Lin)^23^. Yeast were grown in a 100-litre fermenter and extracts were prepared as previously described^7^.

### Affinity purification of pre-A complexes

Uncapped actin pre-mRNA lacking the branch-site adenosine (ΔBS-A *Act* pre-mRNA) was tagged at its 5′-end with three MS2 aptamers and transcribed in vitro using T7 RNA polymerase from a template prepared with the QuikChange II site-directed mutagenesis kit (Agilent). Yeast ΔBS-A pre-A spliceosomal complexes were assembled for 45 min at 23 °C in a 175 ml splicing reaction containing 40% yeast whole-cell extract and 1.8 nM ΔBS-A *Act* pre-mRNA with prebound MBP–MS2 fusion protein. The splicing reaction was subsequently chilled on ice and cleared by centrifugation for 10 min at 9,000 rpm at 4 °C in a Fibrelite F14-14 × 50 cy rotor (Thermo Fisher Scientific). It was then loaded onto two columns, each packed with 600 μl amylose resin (New England BioLabs) that were pre-equilibrated with GK75 buffer (20 mM HEPES-KOH pH 7.9, 1.5 mM MgCl_2_, 75 mM KCl, 5% glycerol, 0.01% NP40, 0.5 mM dithiothreitol (DTT) and 0.5 mM phenylmethylsulfonyl fluoride (PMSF)). The matrix with bound complexes was washed with 3 ml GK75 buffer and spliceosomes were eluted with 15 mM maltose in GK75 buffer. For electron microscopy, peak elution fractions containing approximately 40 pmol of spliceosomal complexes were pooled (1 ml total volume), crosslinked with 0.2 mM BS3 (Thermo Fisher) for 1 h on ice, and loaded onto a 17 ml linear 10–30% (*v*/*v*) glycerol/0–0.1% glutaraldehyde gradient containing GK75 buffer. Samples were centrifuged for 17 h at 24,400 r.p.m. in a SureSpin 630 rotor (Sorvall) and collected manually from the top in 28 fractions of 555 μl each. Crosslinking was stopped by adding 50 mM glycine, pH 7.7, and incubating for 2 h on ice. Fractions were analysed by Cherenkov counting in a Tri-Carb 2100TR scintillation counter (Packard). Two peak fractions containing 2.4 pmol of spliceosomal complexes were buffer-exchanged to GK75 with no glycerol, and concentrated to 250 μl in an Amicon Ultra-0.5 centrifugal filter unit Ultracel-50 (Merck), and then used for preparation of cryo-EM grids. Pre-A complexes assembled on the U257A *Act* pre-mRNA were purified as described above, with the following modifications. The template for in vitro transcription of the U257A *Act* pre-mRNA was purchased from Genscript. Pre-A complexes were assembled in a 252 ml splicing reaction containing 1.7 nM pre-mRNA; before preparation of cryo-EM grids, three peak gradient fractions were buffer exchanged to GK75 buffer containing 0.3% glycerol and samples were concentrated to 100 μl.

### RNA and protein composition of pre-A complexes

To determine the RNA and protein composition of the pre-A complexes, we purified the complexes essentially as described above, except that we used a 12-ml splicing reaction, we washed spliceosomes bound to an amylose matrix with 10 ml GK150 and 10 ml GK75 buffer, and we did not incubate the eluted complexes with BS3. Furthermore, the complexes were fractionated on a 10–30% glycerol gradient lacking glutaraldehyde by centrifugation in a TH660 rotor (Thermo Fisher Scientific) for 16 h at 21,500 r.p.m. RNA and proteins were separated on NuPAGE 4–12% Bis-Tris gels (Invitrogen) and visualized by staining with SYBR Gold (Invitrogen) and Coomassie, respectively. The entire lanes were cut into 23 slices (680 fmol ΔBS-A pre-A) or 16 slices (170 fmol U257A pre-A) and proteins were in-gel digested with trypsin overnight. Resulting peptides were separated on a C18 column using an UltiMate3000 (Dionex) ultrahigh performance liquid chromatography system, and analysed by electrospray ionization mass spectrometry in a Thermo Scientific Q Exactive HF (ΔBS-A pre-A) or Orbitrap Exploris 480 mass spectrometer (U257A pre-A). Data were acquired using Thermo Exactive Series 2.8 SP1 and Orbitrap Exploris 480 3.0 software. The U257A pre-A complex was measured in duplicate, and a sum of both measurements is shown in Supplementary Table 1. Proteins were identified by searching fragment spectra against the *S. cerevisiae* Genomic Database (SGD; https://www.yeastgenome.org) using Mascot v.2.3.02 as a search engine. For immunoblotting, proteins were separated on denaturing 4–12% NuPAGE gels, transferred to Amersham Protran 0.2-μm nitrocellulose membranes (Cytiva), immunostained with an Amersham ECL Western Blotting Detection Kit (Cytiva), and visualized with an Amersham Imager 680 (Cytiva). Antibodies against the yeast Prp5 and Lea1 proteins were provided by S.-C. Cheng.

### Protein–protein crosslinking and identification

For CXMS experiments, spliceosomes were assembled in a 400-ml (experiment 1) or 300-ml (experiment 2) splicing reaction containing 40% yeast whole-cell extract from the 3.2.AID/CRL2101 strain. Following MS2 affinity selection, purified spliceosomal complexes were crosslinked with 250 μM BS3 for 1 h at 8 °C in a total volume of 3 ml. The reaction was split in half and loaded onto two 17-ml 10–30% (*v*/*v*) glycerol gradients and centrifuged in a Surespin 630 rotor (Thermo Fisher Scientific) for 16 h at 24,400 r.p.m. The gradients were fractionated by hand from the top into 28 fractions. Three peak fractions from each gradient, containing approximately 15 pmol of pre-A complexes, were pooled and the crosslinked complexes were pelleted by ultracentrifugation in a S100-AT4 rotor (Thermo Fisher Scientific) and analysed as previously described^24^. After tryptic digestion, peptides were reverse-phase extracted using Sep-Pak Vac tC18 1cc cartridges (Waters) and fractionated by gel filtration on a Superdex Peptide PC3.2/30 column (GE Healthcare). Next, 50 μl fractions corresponding to an elution volume of 1.2–1.8 ml were analysed in triplicate on a Thermo Scientific Q Exactive HF-X (Experiment 1) or Orbitrap Exploris 480 mass spectrometer (Experiment 2) using Thermo Exactive Series 2.9 and Orbitrap Exploris 480 1.1 software, respectively. Protein–protein crosslinks were identified using the pLink 2.3.9 search engine (pfind.ict.ac.cn/software/pLink) and filtered at a false discovery rate (FDR) of 1% or 5% according to the developer’s recommendations^25,26^.

### EM sample preparation and image acquisition

Purified ΔBS-A or U257A pre-A complexes were absorbed for 15 min or 25 min, respectively, to a thin-layer carbon film that was subsequently attached to R3.5/1 Quantifoil grids. Next, 3.8 μl of sample buffer was applied to the grid and excess buffer was removed using an FEI Vitrobot loaded with pre-wetted filter paper, with a blotting force of 7, blotting time of 6.5 s, at 100% humidity and 4 °C. The sample was subsequently vitrified by plunging into liquid ethane. All cryo-EM data of the ΔBS-A pre-A complex and dataset 1 of the U257A pre-A complex were acquired at 300 kV on a FEI Titan Krios electron microscope (Thermo Fisher Scientific), equipped with a Cs corrector. Images were recorded in integration mode at ×120,700 magnification, corresponding to a calibrated pixel size of 1.16 Å at the specimen level, using a Falcon III direct electron detector. Micrographs were recorded via a Thermo Fischer EPU 2.1, using an exposure time of 1.02 s with 40 movie frames and a total dose of 44 e^−^ per Å^2^. In total, 87,604 and 9,170 micrographs were recorded for the ΔBS-A pre-A complex and dataset 1 of the U257A pre-A complex, respectively. Dataset 2 of the U257A pre-A complex (18,332 micrographs) was acquired at 300 kV on an FEI Titan Krios electron microscope (Thermo Fisher Scientific), in integration mode at a calibrated pixel size of 1.06 Å at the specimen level, using a Falcon III direct electron detector. Micrographs were recorded via a Thermo Fischer EPU 2.1, using an exposure time of 1.02 s with 40 movie frames and a total dose of 58 e^−^ per Å^2^.

### Image processing

Frames were dose-weighted, aligned and summed with MotionCor 2.0 (ref. ^27^). The defocus values and equiphase averaging (EPA) of the micrographs were determined using Gctf^28^. Micrographs with a defocus range of 1 μm to 4 μm and a resolution of better than 4.3 Å based on EPA estimation were retained for further processing. For the ΔBS-A pre-A complex, 74,230 out of 87,604 summed micrographs were further processed. Initially, approximately 5.4 million particles were automatically picked using Gautomatch (https://www2.mrc-lmb.cam.ac.uk/research/locally-developed-software/zhang-software/). They were then extracted with a box size of 440 × 440 pixels, and binned to 110 × 110 pixels (pixel size of 4.64 Å) in RELION 3.0 (http://www2.mrc-lmb.cam.ac.uk/relion/index.php/Main_Page). Several iterations of reference-free two-dimensional (2D) classification were performed in RELION 3.0, and ‘bad classes’ showing fuzzy or uninterpretable features were removed, yielding 3,143,491 ‘good particles’. A subset of 308,419 particles was used to generate an initial 3D map using the ab initio reconstruction function in cryoSPARC^29^. Using the ab initio model, this subset of particles was further 3D classified into five classes in RELION 3.0. The best class showing clear features of U1 and U2 snRNP was selected, and the more flexible U2 snRNP region of this map was erased using UCSF Chimera v.1.13.1 (ref. ^30^). The remaining U1 snRNP was low-pass filtered to 35 Å and used as 3D reference for further 3D classification for the entire dataset. The retained 3,143,491 particles after 2D classification of the entire dataset were split into 3 subsets and each subset was 3D classified into 5 classes. The best classes of each subset were combined, yielding 986,393 particles, which were then centred and re-extracted to 200 × 200 pixels (pixel size of 2.32 Å) and further classified into four classes. After 20 iterations of consensus classification (7.5° sampling interval without local search), a mask was placed around the U1 snRNP and the local angular search range was limited to 20° with a finer sampling interval of 3.7°. The best class (with 504,547 particles) was selected, centred and re-extracted with an original pixel size of 1.16 Å with a box size of 400 × 400 pixels and refined with a mask around the U1 snRNP, resulting in a map at 4.3 Å resolution. Next, using the alignment parameters from the aforementioned masked 3D refinement, the 504,547 particles were focus classified with a mask around the high-resolution U1 core, into 4 classes. The best class (containing 226,656 particles) was selected and refined into a map of the entire pre-A complex with an average resolution of 5.9 Å (ranging from roughly 4.5 Å at the U1 region to roughly 15 Å at the U2 region). The U1 and U2 regions were further improved by multibody refinement to 4.1 Å and 10 Å respectively. To further improve the U2 region, we re-extracted the 504,547 particles into a smaller box size of 140 × 140 pixels (pixel size of 2.32 Å) with the U2 snRNP centred, and 3D classified into 5 classes with a mask around the U2 snRNP. The best class, with 160,894 particles, was selected and multibody refined with masks around the U2 5′- and 3′-regions, resulting in a map at 8.3 Å resolution for the 5′-region and one at 9.5 Å resolution for the 3′-region. All the aforementioned resolutions were estimated on the basis of the RELION gold-standard Fourier shell correlation (0.143 criterion).

For the U257A pre-A complex, initially 460,854 and 869,306 particles were extracted from dataset 1 and dataset 2, respectively, and rescaled to 110 × 110 pixels, to the same pixel size of 4.64 Å, in RELION 3.0 (http://www2.mrc-lmb.cam.ac.uk/relion/index.php/Main_Page). After several iterations of reference-free 2D classification, 697,892 ‘good particles’ from the two datasets were combined and classified into four classes by 3D classification with only the U1 part as the starting model, to avoid model bias. No class resembling the structure of the mature A complex was observed. Three classes had no discernible structural features of U1 or U2 snRNPs. One class (of 240,145 particles) clearly exhibited the structure of a pre-A complex, and was selected, centred and re-extracted with a pixel size of 2.32 Å with a box size of 220 × 220 pixels. Re-extracted particles were further 3D classified into four classes with a mask around the U1 part, yielding one class that showed clear secondary structures. This class (80,853 particles) was selected and refined into a map of the entire pre-A complex with an average resolution of 10.4 Å. Multibody refinement improved the U1 part to 7.5 Å. To improve the U2 part, we further classified the 80,853 particles with a mask around the U2 part into four classes, and two classes showing clear U2 density were combined, 3D refined and multibody refined, yielding the U2 part of the structure with 13 Å resolution. All of the aforementioned resolutions were estimated on the basis of the RELION gold-standard Fourier shell correlation (0.143 criterion).

### ΔBS-A pre-A model building and refinement

Templates for the U1 and U2 proteins and RNA were obtained wherever possible from published structures (Extended Data Table 2). The U1 snRNP components, except Prp40, were initially docked as rigid bodies into the 4.1 Å EM map of the U1 region. In the central part of the U1 snRNP (resolution ranging from 3.7 Å to 4.3 Å), side chains were manually adjusted into the map using Coot v.0.8.9.2 (ref. ^31^). The entire model of the U1 snRNP, excluding Prp40, was combined and subjected to real-space refinement in PHENIX v.1.13-2998 (ref. ^32^), with secondary-structure restraints. The solution structure of the Prp40 FF1 domain and the homology model of domains FF2–FF6 predicted by the SWISS-MODEL suite^33^ were truncated to polyalanine, docked into the pre-A map as rigid bodies, and were not refined owing to the limited resolution. The model of Hsh155 (H1–H15) was based on human SF3B1 (H1–H15) but with the sequence changed to that of yeast Hsh155. The model of the ΔBS-A U2/BS helix (U2 nucleotides 32–46; *Act1* pre-mRNA nucleotides 254–268) was generated by deleting the BS-A from the model of the wild-type U2–BS helix using Coot. All U2 snRNP components were docked into the U2 map as rigid bodies without further adjustments, except that Prp9 (amino acids 328–362) and Prp21 (amino acids 173–192) were slightly adjusted using Coot to better fit the EM density, and the linker between the U2–BS helix and SLIIa (U2 nucleotides 47–49) was de novo modelled using Coot. All modelled components in the U2 region were modelled as polyalanine and were not refined owing to the limited resolution. The structural model for the ΔBS-A pre-A complex was fit into the EM density obtained for complexes formed on the U257A mutant *Act1* pre-mRNA. The video showing the structural dynamics seen during the transition from the pre-A to the A complex was generated using ChimeraX v1.1.

### Reporting summary

Further information on research design is available in the Nature Research Reporting Summary linked to this paper.

## Online content

Any methods, additional references, Nature Research reporting summaries, source data, extended data, supplementary information, acknowledgements, peer review information; details of author contributions and competing interests; and statements of data and code availability are available at 10.1038/s41586-021-03789-5.

### Supplementary information

Supplementary Figure 1 PDF showing uncropped images of the gels and western blot shown in this study..

Reporting Summary

Supplementary Table 1 Excel table showing the protein composition of purified *S. cerevisiae* pre-A complexes. Proteins were identified by nano LC-ESI MS using MASCOT as a search engine. Only proteins with a ratio of total peptide spectrum matches (PSMs) to the protein size (expressed in kDa) of at least 1 are shown.

Supplementary Table 2 Excel table showing protein-protein crosslinks identified in *S. cerevisiae* ΔBS-A pre-A complexes. Crosslinks identified by pLink2.3.9 at FDR 1 and 5% are shown. The number of CSMs (crosslinked peptide spectrum matches) are indicated for each crosslinked peptide. “Inter-protein" and “Intra-protein” indicate inter-protein and intra-protein crosslinks, respectively. "Residue 1" and "Residue 2" are the crosslinked residue pairs in Protein 1 and Protein 2, respectively. The data were obtained from two independent crosslinking experiments.

Supplementary Video 1 Mp4 file showing RNP rearrangements during the transformation of the *S. cerevisiae* pre-A complex into an A complex.

## Figures and Tables

**Extended Data Table 1:**
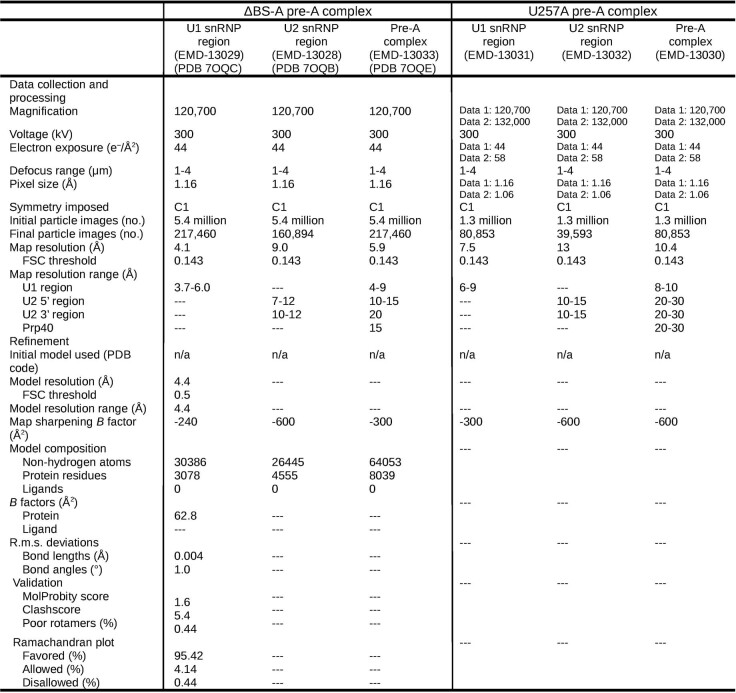
Cryo-EM data collection, refinement and validation statistics

**Extended Data Table 2:**
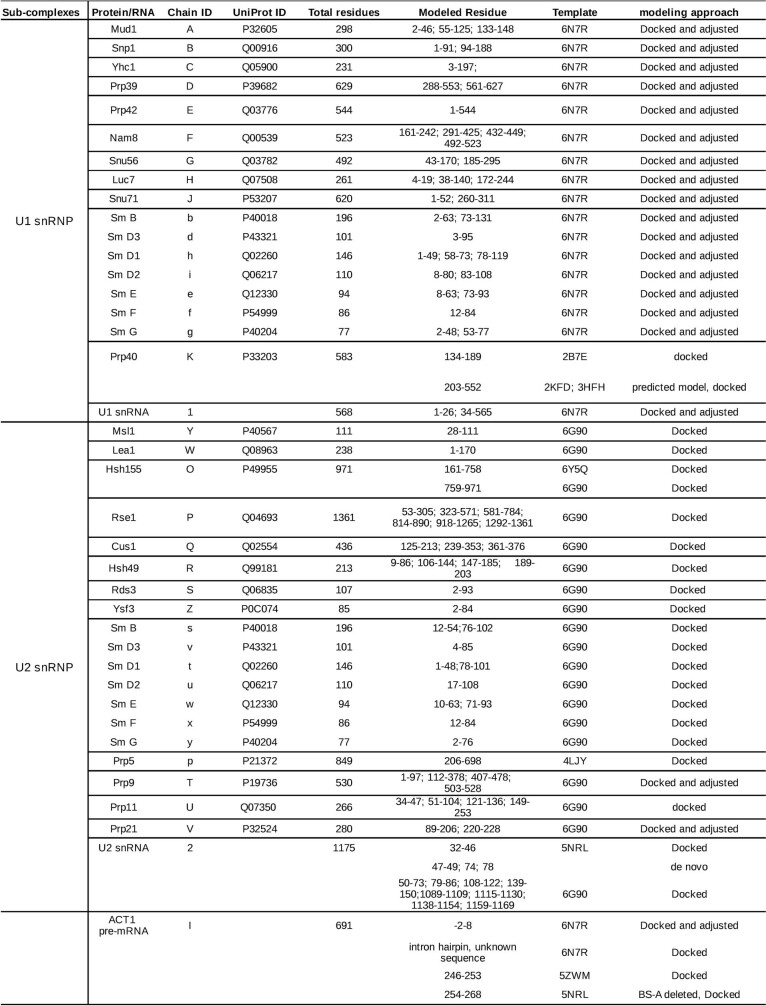
Summary of modelled proteins and RNA in the yeast pre-A structure

RNA and protein regions were modelled and fit into the EM density of the ΔBS-A pre-A complex, as indicated.

## Data Availability

The coordinate files have been deposited in the Protein Data Bank (https://www.rcsb.org) as follows: U1 snRNP region, PDB accession number 7OQC; U2 snRNP region, PDB 7OQB; and composite truncated model of the pre-A complex, PDB 7OQE. The cryo-EM maps have been deposited in the Electron Microscopy Data Bank (https://www.ebi.ac.uk/pdbe/emdb/) as follows: U1 snRNP region of the ΔBS-A pre-A complex, EMD accession number 13029, and of the U257A pre-A complex, EMD 13031; U2 snRNP region of the ΔBS-A pre-A complex, EMD 13028, and of the U257A pre-A complex, EMD 13032; and overall reconstruction of the ΔBS-A pre-A complex, EMD 13033, and of the U257A pre-A complex, EMD 13030. We used the *S. cerevisiae* Genome Database (SGD; https://www.yeastgenome.org) in this study.
