# Supplementary material for: Structural insights into how Prp5 proofreads the pre-mRNA branch site
Source: Nature. 2021 Aug 4;596(7871):296–300. doi: 10.1038/s41586-021-03789-5 (PMC8357632; doi:10.1038/s41586-021-03789-5)
Supplement: Supplementary file 1 — PDF showing uncropped images of the gels and western blot shown in this study.. [file 41586_2021_3789_MOESM1_ESM.pdf]

nature portfolio

https://doi.org/10.1038/s41586-021-03789-5

Supplementary information

Structural insights into how Prp5 proofreads the pre-mRNA branch site

In the format provided by the authors and unedited

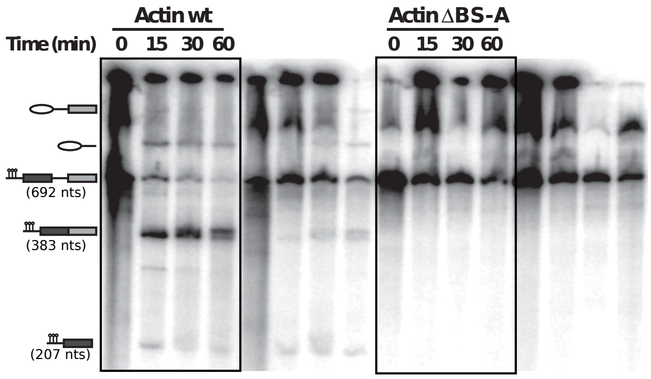

box regions are shown in Extended Data Fig. 1c

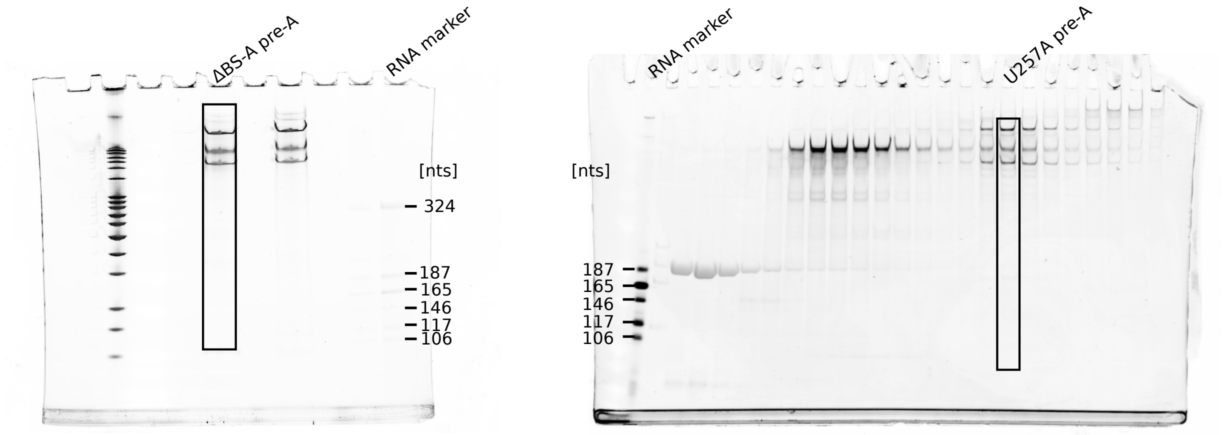

box regions are shown in Extended Data Fig. 1d, left panel

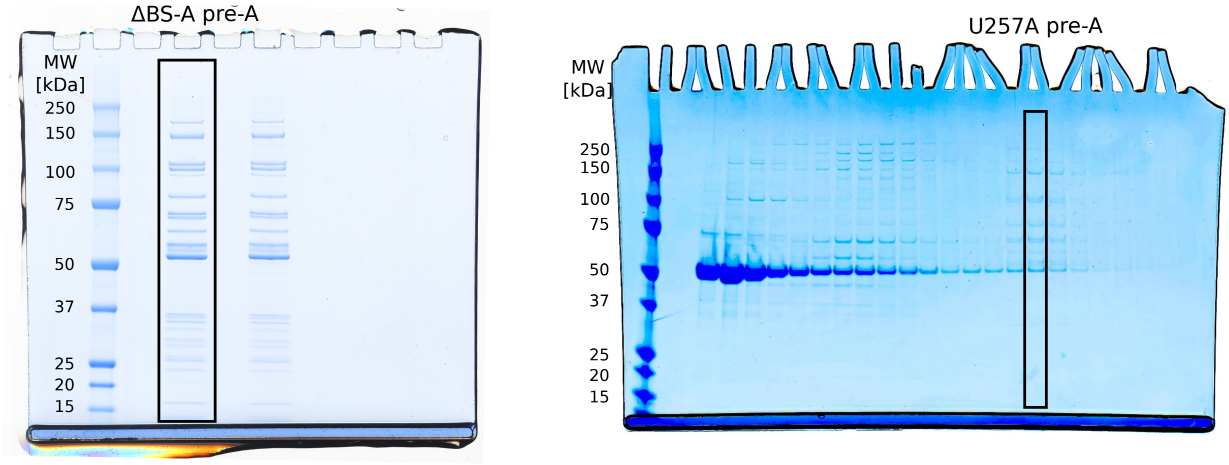

box regions are shown in Extended Data Fig. 1d, right panel

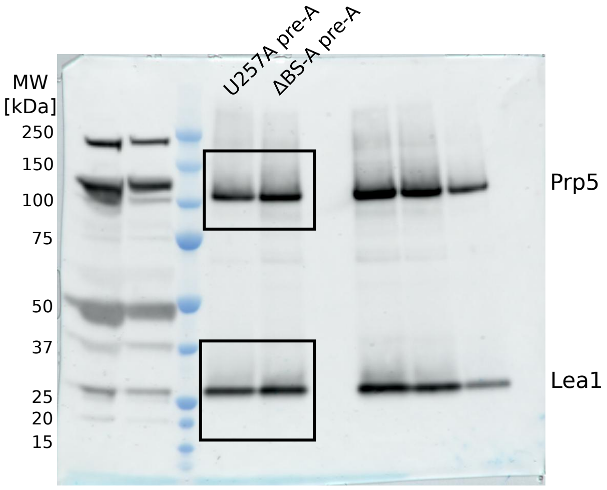

box regions are shown in Extended Data Fig. 1e

Supplementary Figure 1 | Uncropped images of the gels and western blot shown in this study.
