# Supplementary material for: Structural insights into how Prp5 proofreads the pre-mRNA branch site
Source: Nature. 2021 Aug 4;596(7871):296–300. doi: 10.1038/s41586-021-03789-5 (PMC8357632; doi:10.1038/s41586-021-03789-5)
Supplement: Supplementary file 2 — Reporting Summary [file 41586_2021_3789_MOESM2_ESM.pdf]

nature research

Corresponding author(s): Reinhard Lührmann

Last updated by author(s): June 11, 2021

Reporting Summary

Nature Research wishes to improve the reproducibility of the work that we publish. This form provides structure for consistency and transparency in reporting. For further information on Nature Research policies, see Authors & Referees and the Editorial Policy Checklist.

Statistics

For all statistical analyses, confirm that the following items are present in the figure legend, table legend, main text, or Methods section.

n/a	Confirmed	
☐	☒	The exact sample size (n) for each experimental group/condition, given as a discrete number and unit of measurement
☐	☒	A statement on whether measurements were taken from distinct samples or whether the same sample was measured repeatedly
☒	☐	The statistical test(s) used AND whether they are one- or two-sided<br>*Only common tests should be described solely by name; describe more complex techniques in the Methods section.*
☒	☐	A description of all covariates tested
☒	☐	A description of any assumptions or corrections, such as tests of normality and adjustment for multiple comparisons
☒	☐	A full description of the statistical parameters including central tendency (e.g. means) or other basic estimates (e.g. regression coefficient) AND variation (e.g. standard deviation) or associated estimates of uncertainty (e.g. confidence intervals)
☒	☐	For null hypothesis testing, the test statistic (e.g. F, t, r) with confidence intervals, effect sizes, degrees of freedom and P value noted<br>*Give P values as exact values whenever suitable.*
☒	☐	For Bayesian analysis, information on the choice of priors and Markov chain Monte Carlo settings
☒	☐	For hierarchical and complex designs, identification of the appropriate level for tests and full reporting of outcomes
☒	☐	Estimates of effect sizes (e.g. Cohen's d, Pearson's r), indicating how they were calculated

Our web collection on statistics for biologists contains articles on many of the points above.

Software and code

Policy information about availability of computer code

Data collection	Thermo Fischer EPU 2.1, Thermo Exactive Series 2.8 SP1, Orbitrap Exploris 480 3.0
Data analysis	MotionCor v.2, Gctf v.1.06 , Gautomatch v.0.56, RELION 3.0, UCSF Chimera v.1.13.1, ChimeraX v.1.1, cryoSPARC v.2.1, Coot v. 0.8.9.2, SWISS-MODEL suite, pLink v.2.3.9, PHENIX v. 1.13-2998, MASCOT v.2.3.02

For manuscripts utilizing custom algorithms or software that are central to the research but not yet described in published literature, software must be made available to editors/reviewers. We strongly encourage code deposition in a community repository (e.g. GitHub). See the Nature Research guidelines for submitting code & software for further information.

Data

Policy information about availability of data

All manuscripts must include a data availability statement. This statement should provide the following information, where applicable:

- Accession codes, unique identifiers, or web links for publicly available datasets
- A list of figures that have associated raw data
- A description of any restrictions on data availability

The coordinate files have been deposited in the Protein Data Bank as follows: U1 snRNP region (PDB-7OQC), U2 snRNP region (PDB-7OQB) and composite truncated model of the pre-A complex (PDB-7OQE). The cryo-EM maps have been deposited in the Electron Microscopy Data Bank as follows: U1 snRNP region of the ΔBS-A pre-A complex (EMD-13029) and of the U257A pre-A complex (EMD-13031), U2 snRNP region of the ΔBS-A pre-A complex (EMD-13028) and of the U257A pre-A complex (EMD-13032), and overall reconstruction of the ΔBS-A pre-A complex (EMD-13033), and of the U257A pre-A complex (EMD-13030).The S. cerevisiae Genome Database (SGD) was used in this study: https://www.yeastgenome.org.

Field-specific reporting

Please select the one below that is the best fit for your research. If you are not sure, read the appropriate sections before making your selection.

☒ Life sciences ☐ Behavioural & social sciences ☐ Ecological, evolutionary & environmental sciences

For a reference copy of the document with all sections, see nature.com/documents/nr-reporting-summary-flat.pdf

Life sciences study design

All studies must disclose on these points even when the disclosure is negative.

Sample size	No statistical methods were used to predetermine sample size. As many independently recorded images are acquired as part of cryo-EM data collection, the sample sizes are sufficient. For isolation of yeast pre-A complexes, an empirically determined amount of yeast whole cell extract was used to obtain amounts sufficient for the EM and biochemical analyses
Data exclusions	No data were excluded.
Replication	All attempts at replication were successful. Cryo-EM reconstruction inherently contains a high degree of multiplicity arising from being averaged over a large number of independent observations.
Randomization	Samples were not allocated to experimental groups, as this is not a procedure relevant in cryo-EM data processing.
Blinding	Investigators were not blinded during data acquisition and analysis because it is not a common procedure for the methods employed. The methods would not be possible if the investigators were blinded, and furthermore the data processing procedure requires a priori knowledge of the sample to be performed optimally.

Reporting for specific materials, systems and methods

We require information from authors about some types of materials, experimental systems and methods used in many studies. Here, indicate whether each material, system or method listed is relevant to your study. If you are not sure if a list item applies to your research, read the appropriate section before selecting a response.

Materials & experimental systems

n/a	Involved in the study	
☐	☒	Antibodies
☒	☐	Eukaryotic cell lines
☒	☐	Palaeontology
☒	☐	Animals and other organisms
☒	☐	Human research participants
☒	☐	Clinical data

Methods

n/a	Involved in the study	
☒	☐	ChIP-seq
☒	☐	Flow cytometry
☒	☐	MRI-based neuroimaging

Antibodies

Antibodies used	Antibodies against S. cerevisiae Prp5 and Lea1.
Validation	These antibodies were provided by Dr. Soo-Chen Cheng and have been described in a previous publication: Liang & Cheng, Genes Dev. 29, 81-93 (2015).
